# Cardiomyopeptide-Regulated PPARγ Expression Plays a Critical Role in Maintaining Mitochondrial Integrity and Preventing Cardiac Ischemia/Reperfusion Injury

**DOI:** 10.7150/ijms.102763

**Published:** 2025-01-01

**Authors:** Zitong Guo, Geng Qian, Xietian Pan, Yuting Zou, Si Chen, Qinglei Zhu, Zhengju Chen

**Affiliations:** 1 Department of Cardiology, People's Hospital of Xinjiang Uygur Autonomous Region, China.; 2 Department of Cardiology, Chinese PLA General Hospital, the Sixth Medical Center, Beijing, China.; 3 Department of Cardiology, Chinese PLA General Hospital, Beijing, China.; 4 Nanchang Institute of Technology, College of Medicine, China.

**Keywords:** myocardial ischemia-reperfusion injury, PPARγ, cardiomyopeptide, mitochondrial injury

## Abstract

**Background:** Myocardial injury is prone to occur during myocardial ischemia-reperfusion, which further causes adverse cardiac events. Cardiomyopeptide (CMP) has been found to protect the heart against ischemia-reperfusion injury. The present study will explore the molecular and signaling mechanisms associated with the therapeutic effects of CMP.

**Methods:** In this study, the rat myocardial ischemia-reperfusion model was constructed, the pathological changes of myocardial tissues were observed via hematoxylin-eosin (H&E) and Masson staining, and the levels of myocardial injury markers (AST, Mb, TnT) were detected by ELISA. Myocardial tissues of rats in each group were analyzed using transcriptome sequencing (RNA-seq), and the obtained gene expression profiles were analyzed differentially to determine differentially expressed genes (DEGs). In addition, the signaling pathway related to CMP therapy was found by gene set enrichment analysis (GSEA), and PPARγ was detected by qRT-PCR, WB, and IHC staining. The mitochondrial function of myocardial tissues was detected by mitochondrial respiratory chain activity, JC-1, and reactive oxygen species (ROS).

**Results:** Animal assays showed that CMP could significantly improve myocardial injury and reduce the levels of AST, MB and cTnT. RNA-seq analysis results showed that PPARγ signaling pathway is a potential signaling pathway for CMP treatment of myocardial injury in rats. The experimental results showed that CMP can significantly up-regulate PPARγ expression in myocardial tissues, inhibit ischemia reperfusion-induced myocardial injury, and alleviate mitochondrial respiratory disorders.

**Conclusion:** CMP can improve myocardial injury in rats by alleviating mitochondrial respiratory dysfunction and reducing myocardial tissue damage and inflammatory infiltration via the regulation of PPARγ signaling pathway.

## Introduction

The phenomenon known as myocardial ischemia-reperfusion, which involves restoring blood flow to the heart after occlusion of the coronary arteries, is a crucial process aimed at preventing the death of myocardial cells resulting from ischemia 1. In clinical practice, myocardial ischemia-reperfusion has been proposed as a treatment strategy for acute myocardial infarction (AMI) and has shown significant reductions in infarct size, as well as the related morbidity and mortality in real-world scenarios 2. However, it has been observed that the process of reperfusion can cause further damage to ischemic cardiomyocytes, known as myocardial ischemia-reperfusion injury, potentially leading to cardiogenic shock or unfavorable outcomes after circulatory arrest 3. Therefore, it is crucial for the improvement of the prognosis of patients with myocardial infarction to implement preventive and therapeutic strategies to reduce myocardial cell death 4. The pathophysiology of myocardial ischemia-reperfusion injury encompasses a complex series of events, including reduced adenosine triphosphate (ATP) levels, hydrogen ion accumulation, and excessive calcium influx, as well as reactive oxygen species (ROS) generation. These processes ultimately lead to damage and death of myocardial cells 5. Additionally, myocardial ischemia-reperfusion injury involves signaling cascades associated with inflammatory cells and cytokines, triggering an inflammatory response 6.

Based on the understanding of the pathogenesis of myocardial ischemia-reperfusion injury, the use of drugs for cardiac protection is currently an important event for addressing pathological conditions 7. Some polypeptide drugs have been shown to be effective in intervening cardiac dysfunction caused by myocardial ischemia-reperfusion, such as cardiomyopeptides (CMP). CMP is a kind of perioperative myocardial protection adjuvant drug independently developed by Chinese researchers. Its main component is a small molecule extracted from the ventricular muscle of healthy Suckling pigs. Studies have shown that CMP has significant biological activity in many animal experiments and can enhance cardiac function in young rats with ischemia-reperfusion injury 8. A review has shown that CMP can not only reduce the content of myocardial enzymes after myocardial ischemia-reperfusion injury but also inhibit the occurrence of myocardial lipid peroxidation; it can also improve the fluidity of membrane lipids and reduce the deformation and necrosis of myocardial cells 9. Furthermore, the ability of CMP to reduce myocardial damage and ameliorate cardiomyopathy was demonstrated by assessing markers of myocardial damage (such as myocardial enzymes and lipid peroxidation markers) or by light microscopy and electron microscopy. Although the existing evidence has revealed the role of CMP in repairing myocardial damage, further research should be warranted to clarify the mechanism of action. This study, based on animal experiments and transcriptomic analysis, attempts to elucidate the mechanism of CMP affecting myocardial ischemia-reperfusion injury from the molecular level.

The efficacy of CMP in treating myocardial ischemia-reperfusion injury has been reflected in the present available literature. However, there have been no studies on the mechanism by which CMP improves damaged myocardium tissues. This study was based on the establishment of a rat ischemia-reperfusion model to verify the effect of CMP by detecting the pathological changes of myocardial tissues and the content of myocardial injury markers. Combining transcriptome analysis to explore the potential signaling mechanism at the molecular level and verify it through experiments, so as to provide valuable insights for reducing and controlling myocardial ischemia-reperfusion injury in clinical practice.

## Methods

### Animal model establishment and grouping

Eight-week-old male Sprague-Dawley (SD) rats (n=36) were deliberately chosen and subsequently divided into six groups, Control (n=6), Model (n=6), Low-dose CMP (n=6), Mid-dose CMP (n=6), High-dose CMP (n=6) and CMP + GW9662 (n=6) group. Rat models of ischemia-reperfusion injury were established, and the rats were anesthetized with pentobarbital, immobilized on the surgical table, intubated, and mechanically ventilated. Thoracotomy was conducted in the 4th/5th intercostal space of the left sternum. Following rib separation and pericardium removal, the left anterior descending coronary artery was ligated using a 7/0 silk thread and perfusion was sustained for 30 minutes. The untreated Control group was juxtaposed with the Model group, which consisted of rats that had undergone myocardial ischemia-reperfusion injury. The Treatment groups (low-dose, mid-dose group, and high-dose) were separately subjected to tail intravenous injection of CMP (Zhen-Ao pharmaceutical, Dalian, China, 20211001) at doses of 18.9 mg/kg, 56.7 mg/kg, and 170.1 mg/kg. Rats in the CMP+GW9662 group were pretreated with an intraperitoneal injection of PPARγ inhibitor (GW9662, MCE, HY-16578) 5 mg/kg·d, and the middle dose of CMP was injected through the tail vein 14 days after treatment. The treatment groups were treated continuously for 28 days 10. Following the modeling process, the animals were euthanized after 40 days, and subsequent dissection of myocardial tissue was performed 11. The present study was reviewed and approved by the Experimental Animal Welfare and Ethics Committee of the People's Hospital of Xinjiang Uygur Autonomous Region, China, and conducted in accordance with relevant operating guidelines and regulations. The batch number is SYDW2022091101.

### Primary cardiomyocyte extraction

The hearts of newborn rats (within 24h after birth) were extracted and placed in a large dish containing PBS (1:50 double antibody). Cut off the large blood vessels and atria attached to the surface of the heart, and put them into a 5ml sterilized centrifuge tube and fully cut them into slime. Add about 3ml collagenase and 1.5ml 0.05% pancreatic enzyme to thoroughly blow well, and discard the supernatant after digestion 12. About 3ml collagenase and 1.5ml0.05% pancrease were added, and the supernatant was taken after digestion. DMEM containing 10% fetal bovine serum was added into the medium dish. About 3ml collagenase and 1.5ml0.5% pancrease were added into the remaining precipitation, and digested for 10min. Put in the incubator for 2 to 3 hours until the fibroblasts stick to the wall, gently blow the medium, and centrifuge all the medium dishes at 3000 rpm for 5min. The supernatant was discarded, DMEM medium containing 10% calf serum and Brdu (10mM) (1:80) were added and cultured in a medium dish. Cardiomyocytes can be obtained after 48h fluid change. The morphology and pulsation of cardiomyocytes were observed 13.

### Hematoxylin-eosin (H&E) and Masson staining

Rat myocardial tissues were procured and subsequently embedded in paraffin. From these tissues, 4-μm-thick sections were meticulously prepared by a microtome and stained by hematoxylin-eosin for histopathological examination 14. The examination was conducted under a biomicroscope (Olympus, BX53). The staining procedure facilitated the identification of a blue coloration in the nucleus, along with varying degrees of red coloration in the cell pulp, myofibrils, collagen fibers, and red blood cells. Furthermore, the method previously mentioned was employed to obtain slices with a thickness of 4 μm. Subsequently, histopathology was conducted by subjecting the samples to Masson's trichrome staining and examining them under a light microscopy. The staining procedure resulted in the visualization of collagen fibers, mucus, and cartilage exhibiting a blue coloration, whereas cytoplasm, muscle, fibrin, and erythrocytes displayed a red coloration. Additionally, the nuclei appeared as blue-black in color 15.

### Automatic biochemical analyzer test of serum biomarkers of myocardial injury

Peripheral blood samples were collected from rats, and serum was isolated to evaluate myocardial injury markers, specifically serum aspartate aminotransferase (AST), Cardiac troponin T (cTnT), and myoglobin (Mb) 16. Automatic biochemical analyzer (BECKMAN, AU5800) were used to detect these markers under the instructions provided by the manufacturer (COIBO BO, CB10802-Ra, CB10876-Ra, and CB10480-Ra) 17, 18.

### Quantitative real-time polymerase chain reaction (qRT-PCR)

RNA was extracted from the samples by TRIzol kit, and the RNA concentration and purity were determined 18. The RNA was converted to cDNA using reverse transcription kit for qRT-PCR. Taking GAPDH as the internal reference standard, the primers sequence is as follows: PPARγ (F: CTGTTCGCCAAGGTGCTCCA, R: AGGCTCATATCTGTCTCCGTCTTCT), GAPDH, (F: AGGTTGTCTCCTGTGACTTCAA, R: CTGTTGCTGTAGCCATATTCATTG) 19.

### Western blot (WB)

Myocardial tissues were collected, protein lysates were extracted, and total proteins were determined. After being loaded into the SDS-polyacrylamide gel electrophoresis (PAGE), the proteins were transferred to a polyvinylidene fluoride (PVDF) film. The membrane was enclosed in TBST solution for 1 hour and then incubated with PPARγ (Proteintech, 16643-1-AP) at 4℃ overnight. After washing, the second antibody of the membrane was incubated for 1 hour. In this experiment, β-actin (Proteintech, 81115-1-RR) was used as the internal parameter 20.

### Enzyme-linked immunosorbent assay (ELISA)

Peripheral blood samples were collected from rats, and the serum was isolated. The content of mitochondrial respiratory chain complex I was determined by ELISA kit (Gelatins, JLC1921) according to the protocols. Optical density (OD) values at a wavelength of 450 nm were measured using an enzyme marker (BMG LABTECH, SPECTROstar Omega) 21.

### ROS detection

Rat myocardial tissues were collected, single-cell suspension was prepared, and ROS levels were detected. ROS content in myocardial tissues was determined using the kit (Elabscience, E-BC-K138-F) according to the protocols provided by the manufacturer. Flow cytometry was used for detection, and the excitation wavelength was set at 500nm and the detection wavelength was set at 525nm 22.

### Mitochondrial membrane potential (JC-1)

JC-1 dyeing solution was prepared based on the manufacturer's protocols. Cardiomyocytes were collected and re-suspended in 0.5mL cell culture medium 23. 0.5mL JC-1 dyeing solution was added and incubated at 37℃ for 20 minutes. After incubation, the solution was centrifuged at 600g and 4℃ for 3-4 minutes, and the supernatant was discarded. After washing the cells with JC-1 staining buffer (1×) twice, the cells were suspended, centrifuged at 600g and 4℃ for 3-4 minutes, and the supernatant was discarded. It was observed under a fluorescence microscope 24.

### Transcriptome sequencing (RNA-seq)

Rats in the Control (n=3), Model (n=3), and Mid-dose (n=3) groups were subjected to the treatment described in Section 2.1. Following the modeling process, the animals were euthanized, and the myocardial tissues were rinsed with phosphate buffered saline (PBS), flash-frozen in liquid nitrogen, and stored at -80℃ for further analysis. Total RNA was collected and purified from myocardial tissues, and RNA quality was assessed via the Agilent 2100 Bioanalyzer to ensure integrity 25. The mRNA containing the Poly A structure was isolated and subsequently fragmented. A cDNA library was synthesized and library fragments were enriched using principal component analysis (PCA). The quality of the enriched fragments was inspected and detected using a computer analysis 26.

### Differential gene expression analysis

To analyze the transcriptome data, differential gene expression analysis was performed using DESeq2. The criteria for selecting DEGs were established as |log_2_FC| > 1 and P < 0.05. The multi-group comparison volcano plot was performed by R software ggplot2 27.

### Gene set enrichment analysis (GSEA)

GSEA was used to identify signaling pathways or biological processes significantly associated with differentially expressed genes (DEGs) with the Kyoto Encyclopedia of Genes and Genomes (KEGG) database. Pathways with |NES| > 1, NOM p-val < 0.05, and FDR q-val < 0.25 were defined as significantly enriched 28.

### Statistical analysis

The data were calculated by GraphPad Prism 6.0 and expressed as mean ± SD. *P* < 0.05 was considered statistically significant.

## Results

### CMP significantly improves myocardial injury in rats

**Figure 1:**
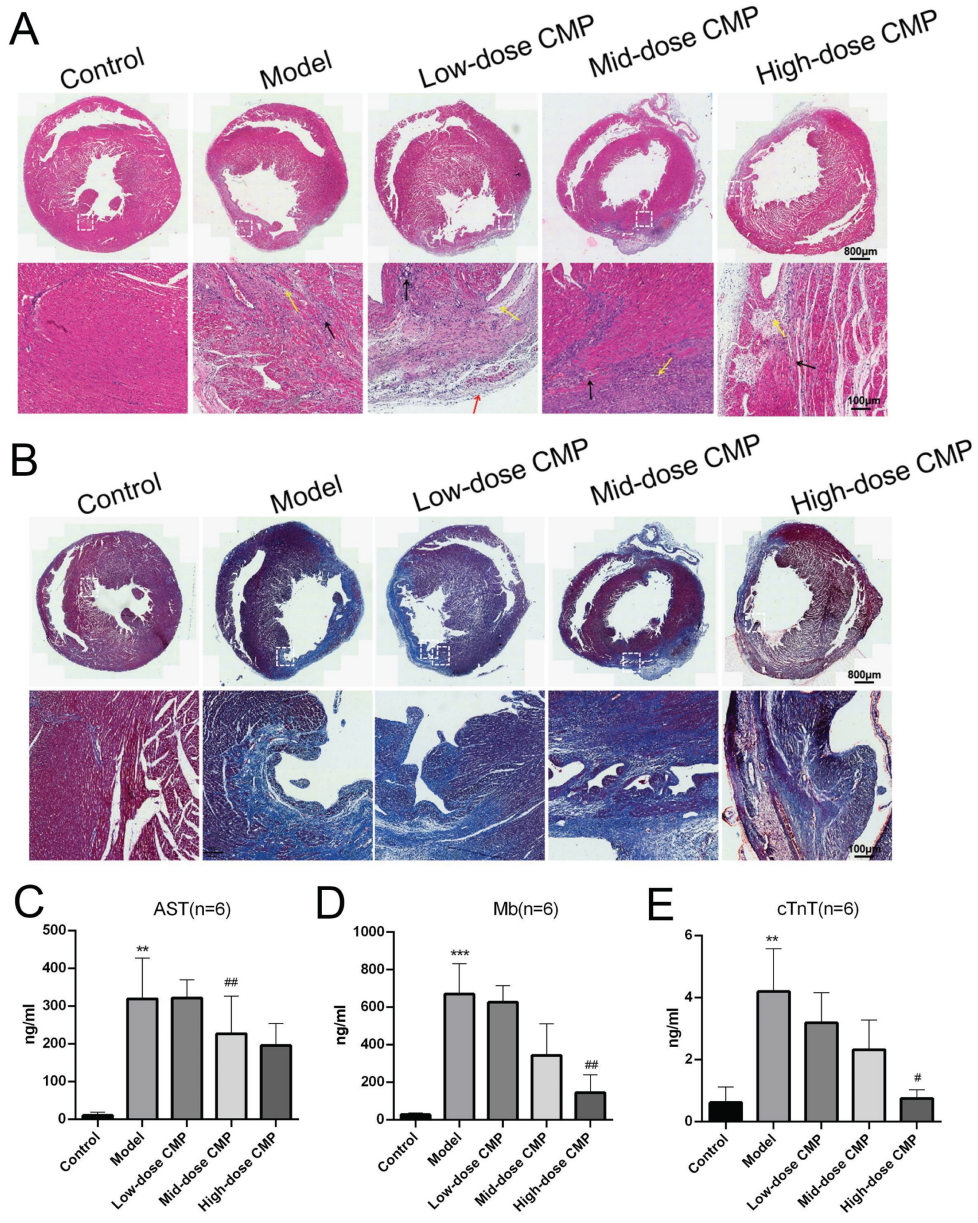
** Therapeutic effect of CMP on myocardial injury.** A) H&E staining to observe the myocardial tissue sections under the treatment of different concentrations of CMP; B) Masson staining to determine the degree of myocardial fibrosis under the treatment of different concentrations of CMP; C-E) The levels of serum markers of myocardial injury (AST, Mb, cTnT) were detected by biochemical analysis.

H&E staining revealed that the myocardial fibers in the Control group were regularly arranged without damage or necrotic gaps. In the Model group, the structure of myocardial fibers was impaired, and the myocardial fibers were broken and dissolved; the muscle space was expanded, and the inflammatory cells were infiltrated. After different doses of CMP treatment, the structure damage and inflammatory infiltration were significantly improved (Figure 1A). Masson staining demonstrated that the myocardial fibers in the Control group were neatly organized, and there were fewer collagen fibers. The degree of myocardial fibrosis and collagen fibers was notably increased in the Model group. After different doses of CMP treatment, myocardial fibrosis was significantly improved and collagen fibers were significantly reduced (Figure 1B). In addition, biochemical analysis found that AST, Mb, and c-TnT levels in the Model group were higher than those in the Control group. In contrast, AST, Mb, and c-TnT levels were reduced after different doses of CMP treatment (Figure 1C).

### PPARγ is a potential target for CMP treatment of myocardial injury in rats

**Figure 2:**
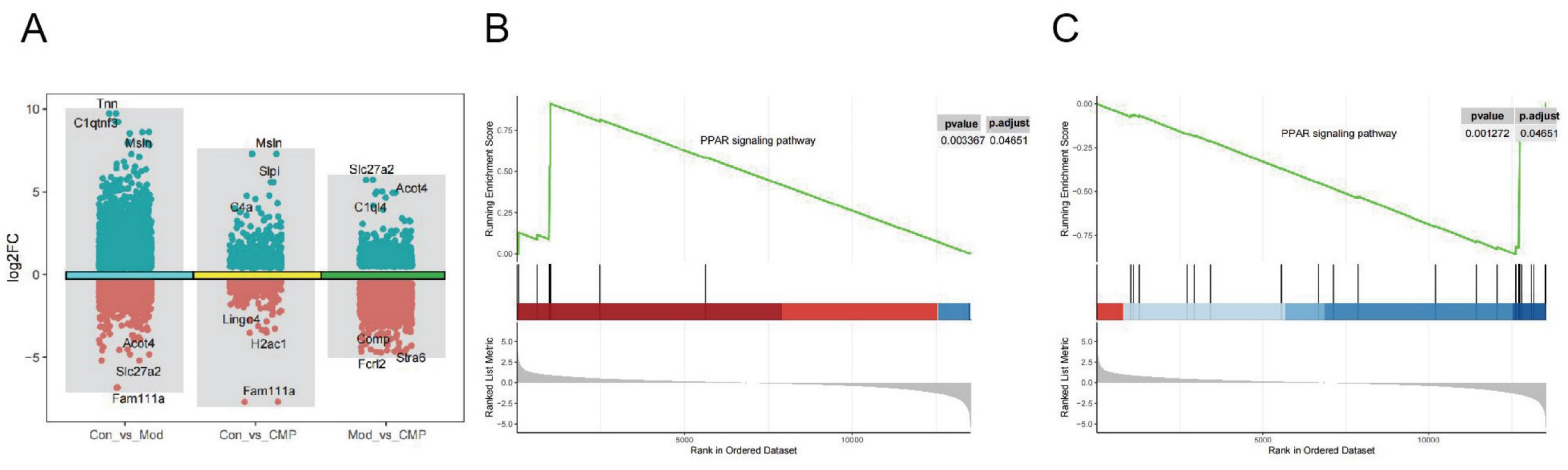
** Identification of pathways related to myocardial injury in rats by transcriptome analysis.** A) The volcano plot showed the gene differential expression of each group; B-C) GSEA results of PPARγ in model-vs-CMP group or control-vs-model group.

Three groups of DEGs (Figure 2A) were obtained by differential gene expression analysis of control, model, and medium-dose CMP. To understand the mechanism of action of CMP, we performed GSEA on control, model, and medium-dose CMP. In both the control-vs-model group and the model-vs-CMP group, an important signaling pathway was found: the PPARγ signaling pathway (figure 2B, 2C). Moreover, PPARγ was activated in the CMP group while suppressed in the Model group.

### CMP does up-regulated PPARγ to improve myocardial injury

**Figure 3:**
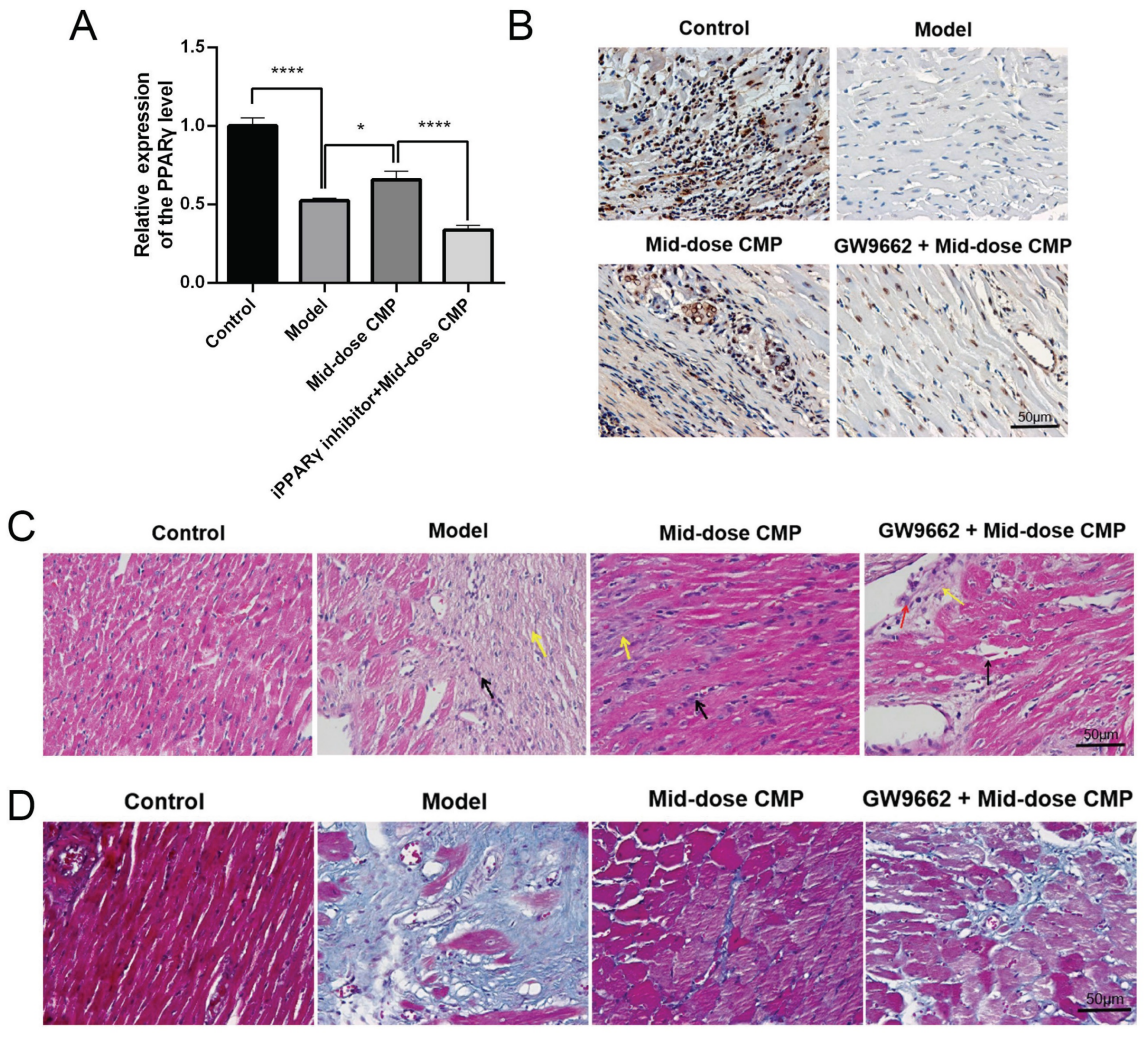
** CMP does up-regulated PPARγ to improve myocardial injury.** A) qRT-PCR to detect PPARγ mRNA levels in each group; B) IHC to detect PPARγ protein levels in each group; C) H&E staining to detect the myocardial tissue injury of rats in each group; D) Masson staining to detect the myocardial tissue injury of rats in each group. * *P* < 0.05, **** *P* < 0.0001.

Based on the therapeutic effects of various concentrations of CMP on myocardial injury in rats, a medium dose of CMP was selected for subsequent experiments in the present study. qRT-PCR results revealed that PPARγ was significantly higher in the Model group than in the Control group; after CMP treatment, there was a significant increase in the level of PPARγ, while in the CMP+ GW9662 group it was significantly suppressed (Figure 3A). This finding was also proved by the IHC assay (Figure 3B). In addition, the results of HE and Masson staining showed that CMP did significantly improve the myocardial fiber damage and inflammatory cell infiltration in rats, but when the GW9662 was used, the degree of myocardial injury was significantly aggravated. (Figure 3C-D).

### CMP regulates PPARγ to alleviate myocardium tissue mitochondrial dysfunction

**Figure 4:**
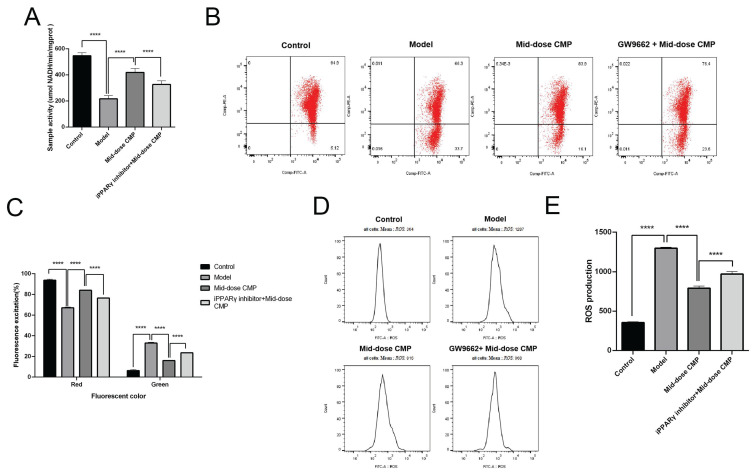
** CMP regulates PPARγ to alleviate myocardium tissue mitochondrial dysfunction.** A) The activity of mitochondrial respiratory complexes was detected in each group; B) The level of mitochondrial membrane potential in each group was determined by flow cytometry; C) The level of mitochondrial membrane potential in each group, red is JC-1 polymer in high potential state, green is JC-1 monomer in low potential state; D) The content of cellular ROS in each group was determined by flow cytometry; E) The cellular ROS content in each groups. **** P < 0.0001.

By detecting the mitochondrial respiratory chain activity of rat cardiomyocytes, it can be seen that myocardial injury causes mitochondrial respiration disorder in rats, but CMP can improve mitochondrial respiration (Figure 4A). JC-1 is a molecular probe for detecting mitochondrial membrane potential, which can reflect early apoptotic events. It showed that the CMP could significantly improve the decrease of mitochondrial membrane potential caused by myocardial injury (Figure 4B, C). In addition, ROS content in injured cardiomyocytes increased rapidly, while ROS level was significantly decreased after CMP treatment (Figure 4D, E). After adding GW9662, the function of CMP in improving mitochondrial dysfunction in myocardial tissue no longer exists. These results suggested that CMP through regulates PPARγ to alleviate myocardium tissue mitochondrial dysfunction.

## Discussion

With more than 800,000 new or recurrent myocardial infarctions each year, it is particularly important to find appropriate treatment and to study its functional mechanisms 9, 29. In this study, a rat myocardial ischaemia‒reperfusion injury model was constructed, and the experiment consisted of normal, model and treatment groups (high, medium, and low doses of CMP for injection) 30, which were used to study the therapeutic effects of CMP on myocardial ischaemia‒reperfusion injury. Both H&E staining and Masson staining showed that the Model group had severe myocardial tissue destruction, inflammatory cell infiltration, and significant myocardial fibrosis, while these manifestations were significantly improved to different degrees in the Treatment group. In addition, the changes in serum markers of myocardial injury also reflected the therapeutic efficacy of CMP on myocardial ischaemia‒reperfusion injury in rats 31.

Inflammation is an important mechanism related to myocardial ischaemia‒reperfusion injury, and myocardial injury from ischaemia‒reperfusion can be improved by inhibiting the inflammatory response 32. In addition, in cardiac tissue, mitochondria, which are present in large numbers, can continuously produce reactive oxygen species (ROS), which are key factors in ischaemia‒reperfusion injury 33, 34. Mitochondrial dysfunction and apoptosis are the most typical features of inflammation-related myocardial injury, which has been identified in lipopolysaccharide (LPS) -induced cardiomyocytes 35. In contrast, fatty acids are the main metabolic substrate of cardiac mitochondria, and inhibition of fatty acid oxidation can attenuate myocardial injury due to excessive ROS 36, 37. Peroxisome proliferator-activated receptors (PPARs), members of the nuclear hormone receptor superfamily, have a vital role in regulating energy metabolism 38, 39. PPAR family genes are expressed in multiple tissues, and there is evidence that PPARγ is significantly associated with cardiovascular disease and exerts a significant protective effect on the heart 40, 41.

PPARγ can facilitate the treatment of myocardial ischemia-reperfusion injury 42. For example, rosuvastatin promotes the viability of cardiomyocytes, inhibits the release of LDH, and reduces the production of ROS and the levels of Caspase-9 and cytochrome C to protect primary cardiomyocytes against ischemia-reperfusion injury by upregulating PPARγ and uncoupling protein 2 (UCP2) 43. However, statins such as rosuvastatin are prone to side effects such as muscle injury or muscle inflammation in patients 44, and animal experiments have shown that PPARγ receptor agonists alone can increase the risk of bladder cancer in rats 45. In addition, CMP, the main component of which is a polypeptide active substance extracted from the ventricle of suckling pigs, is one of the important substances involved in maintaining the stability of physiological pH in cardiac myocytes. It can scavenge reactive oxygen species and other free radicals, thus preventing and protecting myocardial injury caused by various factors such as cardiac ischemia-reperfusion 46.

Small molecule peptides have a simple structure, unsaturated, small molecular weight, unique physiological activities, and health care effects that the original protein and monomer amino acids do not have, and have triple functions of nutrition, conditioning, and adjuvant therapy 47. The human body absorbs proteins in the form of peptides. For example, in the intestine, proteins are enzymatically hydrolyzed by digestive enzymes, and small molecular peptides can be completely absorbed and enter the blood circulation system, that is, they can enter cells in their original form through osmosis and be absorbed and utilized by cells 48. However, some scholars believe that the adjuvant use of small molecular peptides will increase the burden of patients 49. For example, thymic peptides contain sensitizing ingredients of animals, which may cause skin rash, fever, chest tightness, palpitations, dyspnea, headache and other allergic symptoms 50. In 2005, myocardin was approved by the National Food and drug administration as a class of chemical drugs for the adjuvant drug of perioperative myocardial protection in cardiac surgery 51. But so far, the therapeutic mechanism of CMP on myocardial ischemia-reperfusion injury remains to be explored. This study intends to explore whether CMP mediates the protective effect of PPARγ on myocardial ischemia-reperfusion injury by combining the regulatory effect of myocardial peptide on PPARγ. In conclusion, CMP can improve myocardial injury in rats by alleviating mitochondrial respiratory dysfunction and reducing myocardial tissue damage and inflammatory infiltration via the up-regulation of PPARγ signaling pathway.
